# Burden of drug use disorders in the United States from 1990 to 2021 and its projection until 2035: results from the GBD study

**DOI:** 10.1186/s12889-024-19142-0

**Published:** 2024-06-19

**Authors:** Tongchao Zhang, Lin Sun, Xiaolin Yin, Hui Chen, Lejin Yang, Xiaorong Yang

**Affiliations:** 1 Clinical Epidemiology Unit, Qilu Hospital of Shandong University, 107 Wenhuaxi Road, Jinan, Shandong 250012 China; 2 Clinical Research Center of Shandong University, 107 Wenhuaxi Road, Jinan, Shandong 250012 China; 3 Department of Pharmacy, Qilu Hospital of Shandong University, 107 Wenhuaxi Road, Jinan, Shandong 250012 China; 4 Department of Psychology, Qilu Hospital of Shandong University, 107 Wenhuaxi Road, Jinan, Shandong 250012 China

**Keywords:** Global Burden of Disease, Incidence, Prevalence, Death, Disability-adjusted life years, Drug use disorders, The United States, Trends, Projection, Epidemiology

## Abstract

**Background:**

Drug use disorders (DUDs) have emerged as one of the most significant public health crises, exerting a substantial influence on both community health and socio-economic progress. The United States (US) also suffers a heavy burden, it is necessary to figure out the situation from multiple perspectives and take effective measures to deal with it. Therefore, using the data from the Global Burden of Diseases, Injuries, and Risk Factors (GBD) 2021, we evaluated this topic.

**Methods:**

Annual data on DUDs-related burden were collected from the GBD study 2021. We calculated the indicator of estimated annual percentage change (EAPC) to evaluate the changing trend of burden. The Bayesian model for age-period-cohort was introduced to forecast the burden.

**Results:**

In 2021, the number and age-standardized rate of prevalence were particularly prominent, with 12,146.95 thousand and 3821.43 per 100,000, respectively. Higher burden was also observed in males, 15–45 years old populations, and opioid use disorders subtype. From 1990 to 2021, the DUDs-related burden increased in the US and all states, especially in West Virginia; and the national death-related burden with the highest increase (EAPC = 7.96). Other significant inverse associations were seen between EAPC, age-standardized rates, and socio-demographic index (SDI). Moreover, in the next 14 years, the projected DUDs burden remains exigent.

**Conclusions:**

The burden of DUDs in the US is heavy and has been enlarging. This study proposes that greater attention should be paid to the strategies in males, the younger population, opioid use disorders, and low-SDI states implemented by decision-makers to achieve goals such as reducing burden.

**Supplementary Information:**

The online version contains supplementary material available at 10.1186/s12889-024-19142-0.

## Background

Drug use disorders (DUDs), refer to the forced and endless use of certain drugs with dependency characteristics in the pursuit of special psychological effects rather than medical purposes, can cause serious psychological, physiological consequences, and social problems [1–3], including cognitive impairment, suicidal tendencies, decreased quality of life, and risk of infectious diseases [4–7]. DUDs continue to be a heavy burden globally, according to the Global Burden of Disease, Injuries, and Risk Factors Study (GBD) 2019, DUDs were in the top 20 leading causes of disability-adjusted life years (DALYs) in the 10–49-year age groups in 2019 [8]. Meanwhile, the World Drug Report 2023 indicated that, in 2021, over 296 million people worldwide used drugs; and the number of people suffering from DUDs has soared to 39.5 million, an increase of 45% in the past 10 years [9]. However, only one-fifth of people with drug-related disorders received medication treatment, and the gap in access to treatment among regions continued to widen [9].

Notably, the United States (US) is one of the countries with a heavy DUDs-related burden [1, 8]. According to the GBD study, in 2019, over half of the death cases due to DUDs worldwide occurred in the US [1, 8]. The GBD database has the advantage of systematic analysis and integration of global disease and health data, and the adoption of the GBD data can provide policymakers, researchers, and the public with comprehensive insights into the status of DUD in the US. Therefore, in this study, we retrieved detailed data on the latest burden of DUDs from the GBD study (2021) to fully investigate the magnitude and temporal trends of burden due to DUDs in the US from 1990 to 2021 by age, sex, state, and drug categories; and attempted to predict the burden in the next 14 years. Our results can be helpful for the country to develop more effective population-specific policies and methods.

## Materials and methods

### Data collection and case definition

Similar to the GBD study 2019, the data collection, processing, and overall analysis methods of the GBD study (2021) have been reported in detail previously [10–12]. We collected the data on DUDs-related burden from the Institute for Health Metrics and Evaluation (IHME, https://vizhub.healthdata.org/gbd-results/). We obtained the data on DUDs-related burden about the numbers (in thousands), rates (per 100,000 population), and age-standardized rates (per 100,000 population) of incidence, prevalence, deaths, and DALYs by age, sex, and state from 1990 to 2021. Meanwhile, the information about the socio-demographic index (SDI, an indicator that was calculated based on the fertility rate, education, and income) for the states was also collected [10–12].

In the GBD study (2021), the DUDs were defined based on the Diagnostic and Statistical Manual of Mental Disorders (DSM-IV-TR) or the International Classification of Diseases (ICD-10) diagnostic criteria, including opioid use disorders, cocaine use disorders, cannabis use disorders, amphetamine use disorders, and other DUDs [10, 11]. Other DUDs included hallucinogen dependence, inhalant or solvent dependence, sedative dependence, tranquiliser dependence, and other medicines, drugs, substance dependence [10].

### Evaluation of DUDs-related burden

The prevalence, incidence, mortality, and burden of DUDs (by age, sex, year, and subtype) were generally evaluated in the following ways: Firstly, based on the input raw data of vital registration, verbal autopsy, and surveillance databases, a cause of death database was generated through standardization, ICD mapping, age-sex division, garbage code redistribution, and noise reduction [10, 11]. Secondly, based on this cause of death database, the estimation of DUDs-related burden followed the general Cause of Death Ensemble model (CODEm) strategy [10, 11]. The CODEm strategy mainly included two models: linear mixed-effects regression (LMER) models and spatiotemporal Gaussian process regression (ST-GPR) [10, 11]. With the progress of the CODEm strategy (covariates adjusted), the mortality rate of DUDs and the years of life lost (YLLs) were primarily evaluated [10, 11]. In the GBD 2021 study, the covariate included additional data and increased time smoothing, which improved the stability of the results, especially for the estimates of the US and Western Europe [10, 11]. Thirdly, based on the cause of death database and systematic reviews of epidemiological survey data, using the Bayesian meta-regression method DisMod-MR 2.1, the prevalence, incidence, and years of life lived with disability (YLDs, fully considering the disability weights and comorbidity) was further generated by age, sex, year, and subtype [10, 11]. DisMod-MR 2.1 is a Bayesian meta-regression tool, which gathers data points across multiple sources and accounts for recognized variability factors such as disparities in case definitions and sampling methods to generate internal consistent estimates [10, 11]. And lastly, the DALYs (by age, sex, year, and subtype) were obtained by adding YLLs and YLDs [10, 11]. Based on the global standard population of the GBD study (2021), age-standardized rates were also estimated [13].

### Statistical analysis

Data were described as absolute numbers with 95% uncertainty intervals (UIs) by age, sex, year, state, and drug categories. 95% UI was generated by the 2.5th and 97.5th percentiles of the ordered estimate values for the CODEm process of 1,000 draws [11]. When assessing the temporal trends, we introduced the indicator of estimated annual percentage change (EAPC) [14–16]. The EAPC was calculated based on a regression model, that is, [ln (age-standardized rate) = α + β × (calendar year) + ε]. The EAPC and its 95% confidence interval (CI) were calculated from the model of [100 × (exp (β)-1)] [14–16]. The trends were recognized as a decrease when the upper boundary of 95% CI of EAPC was < 0; while if the lower boundary of 95% CI of EAPC was > 0, the upward trends of burden were defined; otherwise, the trends represented stable [14–16].

Meanwhile, using the Pearson or Spearman rank test, associations between EAPC, DUDs-related burden, and SDI were determined, and the expected associations between them were estimated using the locally weighted regression (LOESS) model [17]. Additionally, we implemented the Bayesian model for age-period-cohort (BAPC) to forecast the burden until 2030 [18–20]. The BAPC model is expressed as n_ij_ = log(λ_ij_) = μ + α_i_ + β_j_ + γ_k_, where λ_ij_ denotes the count of cases, μ denotes the intercept, and α_i_, β_j_, and γ_k_ signify the effect of age, period, and cohort, respectively [18–20]. To account for overdispersion, we utilized the BAPC model, which was implemented using the INLA and BAPC packages in R software [18–20]. The predicted population was obtained from the World Population Prospects 2022 [21].

All statistical analyses and visualization of results were conducted using the R software (Version 4.0.3; https://www.R-project.org/), and the two-tailed *P* value < 0.05 was considered statistically significant.

## Results

### The burden of DUDs in the US in 2021 along with the temporal trend

**Fig. 1:**
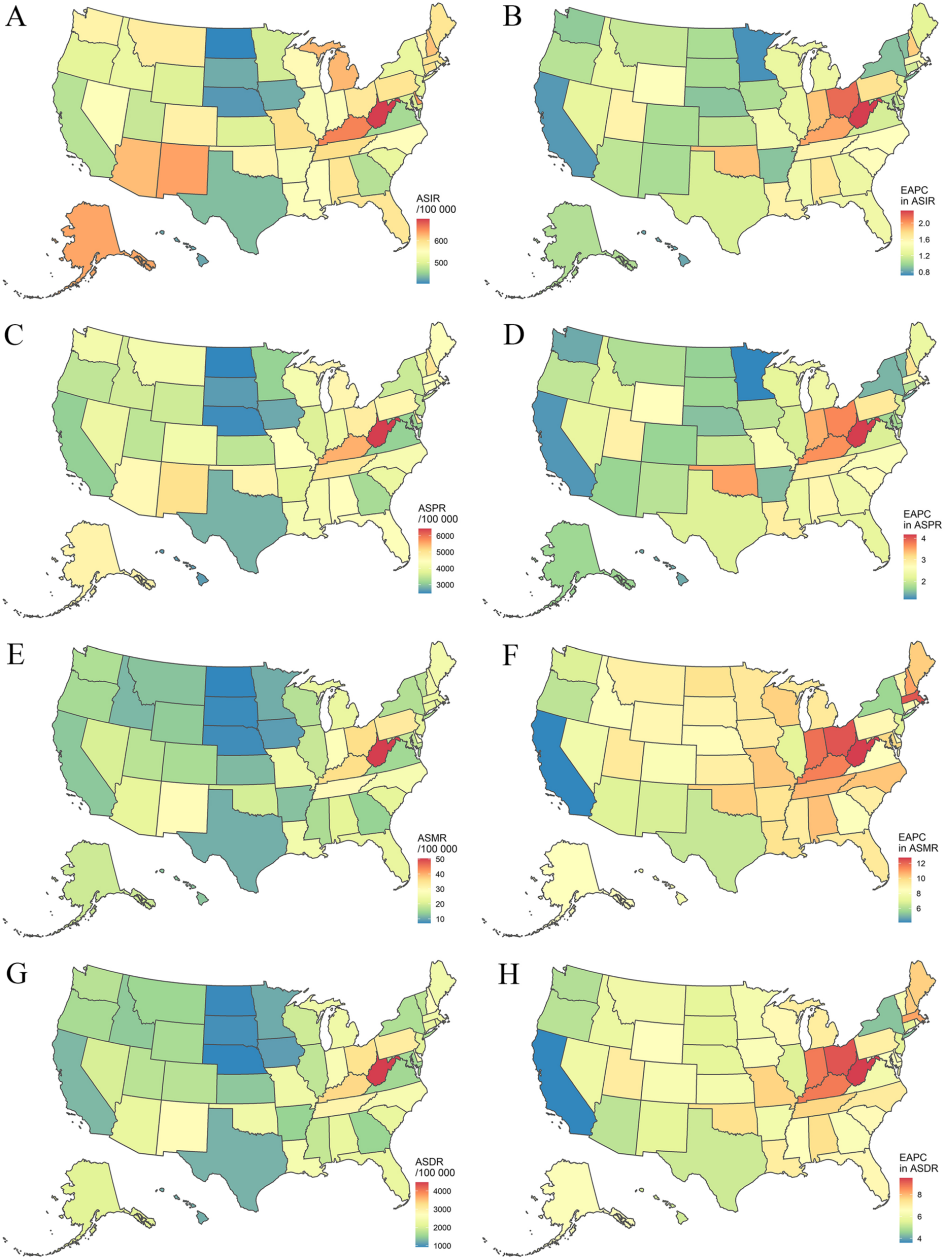
Age-standardized rates of drug use disorders-related burden in 2021 and the temporal trends from 1990 to 2021 by states in the US. **A** ASIR in 2021; **B** EAPC in ASIR from 1990 to 2021; **C** ASPR in 2021; **D** EAPC in ASPR from 1990 to 2021; **E** ASMR in 2021; **F** EAPC in ASMR from 1990 to 2021; **G** ASDR in 2021; and **H** EAPC in ASDR from 1990 to 2021. ASIR: age-standardized incidence rate; ASPR: age-standardized prevalence rate; ASMR: age-standardized mortality rate; ASDR: age-standardized DALYs rate; EAPC: estimated annual percentage change

Nationally, in 2021, the numbers of DUDs-related incident cases, prevalent cases, deaths, and DALYs were 1583.45 (95%UI: 1384.48, 1793.91), 12,146.95 (95%UI: 11,024.58, 13,461.04), 70.89 (95%UI: 64.05, 78.96), and 6484.69 (95%UI: 5471.72, 7481.32) thousand, respectively; accounting for 11.64%, 22.87%, 51.64%, and 41.67% of global numbers, respectively (Additional File 1: Tables S1-S8). Meanwhile, the age-standardized rates of incidence, prevalence, mortality, and DALYs (ASIR, ASPR, ASMR, and ASDR) of the DUDs were 531.19 (95%UI: 462.11, 605.02), 3821.43 (95%UI: 3450.13, 4257.62), 19.52 (95%UI: 17.73, 21.61), and 1944.08 (95%UI: 1632.99, 2249.41) per 100,000, respectively (Additional File 1: Tables S1-S4). It could be observed that the ASIR, ASPR, ASMR, and ASDR were significantly higher in the US than those in the world (3.14 times, 5.76 times, 11.83 times, and 10.18 times, respectively) (Additional File 1: Tables S1-S8). At the state level, the highest absolute number of DUDs-related burden was observed in California, Texas, Florida, New York, Pennsylvania, and Ohio also observed higher numbers (Additional File 1: Tables S1-S4). Meanwhile, West Virginia had the highest age-standardized rates, followed by Kentucky (Additional File 1: Tables S1-S4; Fig. 1A, 1C, 1E, and 1G). From 1990 to 2021, the ASIR, ASPR, ASMR, and ASDR increased in the US, with EAPCs of 1.27 (95%CI: 1.05, 1.48), 2.17 (95%CI: 1.81, 2.53), 7.96 (95%CI: 7.64, 8.29), and 6.12 (95%CI: 5.83, 6.40), respectively (Additional File 1: Tables S1-S4). While a slight increase was only observed in ASMR worldwide (Additional File 1: Tables S5-S8). It could be observed that the burden of DUDs increased in all states from 1990 to 2021, with the highest growth in West Virginia (Additional File 1: Tables S1-S4; Fig. 1B, 1D, 1F, and 1H). The burden and changing trend of DUDs for each sex and type of DUDs was presented in Table S10 (Additional File 1), generally, there was no particularly significant difference from the overall estimates.

### Burden and temporal trends of DUDs in the US by age and sex

**Fig. 2:**
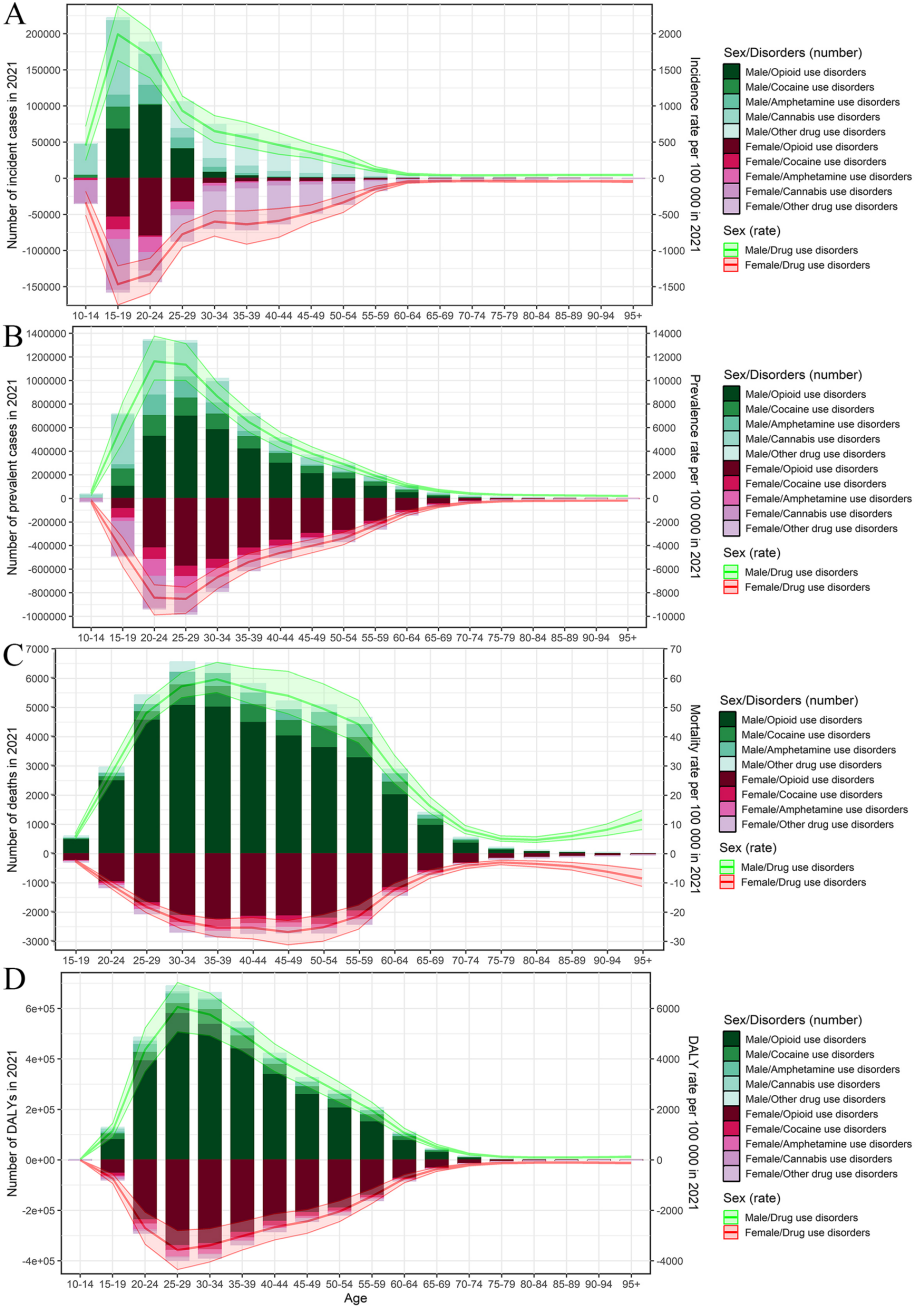
Age-specific numbers and rates of drug use disorders-related burden by sex and subtype in 2021 in the US. **A** age-specific numbers and rates of incidence; **B** age-specific numbers and rates of prevalence; **C** age-specific numbers and rates of deaths; and **D** age-specific numbers and rates of DALYs. The bar plots represented the numbers; the line plots and their shade represented the rates and their 95%UIs. DALYs: disability-adjusted life years; UI: uncertainty interval

In 2021, the DUDs-related burden (both numbers and age-standardized rates) in males was higher than that in females in both the US and the world (Additional File 1: Tables S1-S8; Fig. 2; and Additional File 2: Figure S1). From 1990 to 2021, in the US, the ASIR, ASPR, ASMR, and ASDR increased in both sexes, especially in females (Additional File 1: Tables S1-S4; Additional File 2: Figure S1). However, at the global level, in both sexes, the ASIR and ASPR decreased, while the ASMR and ASDR remained stable, respectively (Additional File 1: Tables S5-S8).

**Fig. 3:**
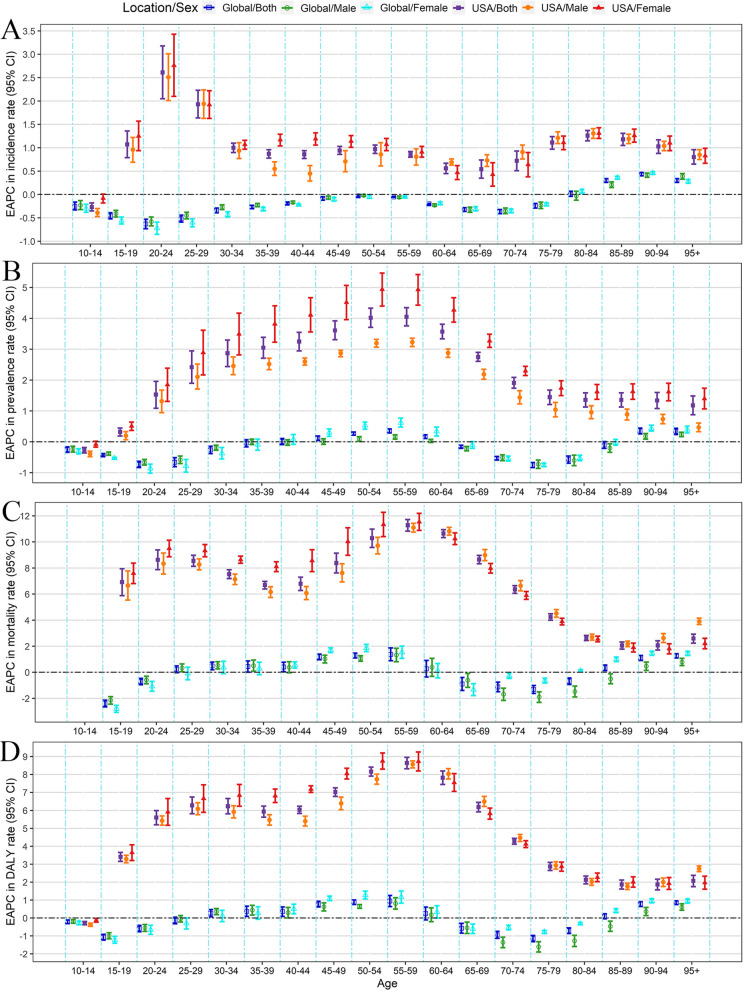
The age-specific changing trends in drug use disorders-related burden by sex from 1990 to 2021 in the US and the world. **A** age-specific EAPCs of incidence rates; **B** age-specific EAPCs of prevalence rates; **C** age-specific EAPCs of death rates; and **D** age-specific EAPCs of DALYs rates. The error bar represented the EAPCs and their 95%CIs. CI: confidential interval; DALYs: disability-adjusted life years; EAPC: estimated annual percentage change

In 2021, similar to the world, more than half of the burden (numbers and age-specific rates) of DUDs was generally concentrated in the population 15–45 years old in the US in both sexes (Additional File 1: Tables S1-S8; Fig. 2; and Additional File 2: Figures S2-S3). Notably, the burden of deaths was still heavy in the population 45–65 years old in both sexes at the US and global levels (Additional File 1: Tables S3 and S7; Fig. 2; and Additional File 2: Figures S2-S3). From 1990 to 2021, in the US, the age-specific rates of burden increased in both sexes in all age-specific groups, except for the < 15 age-specific group (EAPC for ASIR = -0.27, 95%CI: -0.35, -0.18; EAPC for ASPR = -0.27, 95%CI: -0.36, -0.18; and EAPC for ASDR = -0.28, 95%CI: -0.37, -0.19, respectively) (Additional File 1: Tables S1-S4; Fig. 3; and Additional File 2: Figure S3). However, at the global level, in both sexes, the age-specific rates of incidence increased in the > 85 age-specific groups, while the upward trends of age-specific rates of prevalence, deaths, and DALYs were generally observed in the 45–60 and > 90 age-specific groups (Additional File 1: Tables S5-S8; Fig. 3; and Additional File 2: Figure S3).

### Burden and temporal trends of DUDs in the US by drug categories

For the subtypes of DUDs, in 2021, opioid use disorders generally occupied the highest burden in both sexes in the US (except for the incidence burden); the numbers of incident cases, prevalent cases, deaths, and DALYs were 435.11 (95%UI: 362.57, 528.38), 6607.64 (95%UI: 5815.22, 7532.22), 55.45 (95%UI: 48.82, 62.98), and 5317.91 (95%UI: 4382.17, 6157.94) thousand, respectively; the corresponding ASIR, ASPR, ASMR, and ASDR were 151.84 (95%UI: 125.79, 184.96), 2014.62 (95%UI: 1761.65, 2308.32), 15.37 (95%UI: 13.62, 17.33), and 1594.63 (95%UI: 1308.06, 1849.82) per 100,000, respectively (Additional File 1: Tables S1-S4; Fig. 2; and Additional File 2: Figure S1). Similar results could be found at the global level, except for the incidence and prevalence burden (Additional File 1: Tables S5-S8). Moreover, the burden of opioid use disorders accounted for a higher proportion among young people (< 30 years old) (Fig. 2).

From 1990 to 2021, the ASIR remained stable in subtypes of amphetamine (EPAC = -0.02, 95%CI: -0.75, 0.73) and cocaine (EPAC = -0.08, 95%CI: -0.31, 0.14) use disorders, increased in opioid (EPAC = 6.05, 95%CI: 5.44, 6.67) and other drug (EPAC = 0.89, 95%CI: 0.81, 0.96) use disorders subgroups, and slightly decreased in the cannabis (EPAC = -0.04, 95%CI: -0.08, 0.00) use disorders subtype (Additional File 1: Table S1; Additional File 2: Figure S1). The ASPR remained stable in subtypes of amphetamine (EPAC = -0.05, 95%CI: -1.02, 0.93), and decreased in the cannabis (EPAC = -0.05, 95%CI: -0.08, -0.02) use disorders subtype; while increased in cocaine (EPAC = 0.44, 95%CI: 0.26, 0.61), opioid (EPAC = 6.73, 95%CI: 6.21, 7.25), and other DUDs (EPAC = 2.70, 95%CI: 2.55, 2.85) subtypes (Additional File 1: Table S2; Additional File 2: Figure S1). The ASMR increased in all subtypes of DUDs, with the highest EAPC of 11.32 (95%CI: 10.50, 12.16) in the amphetamine use disorders subtype (Additional File 1: Table S3; Additional File 2: Figure S1). Except for the cannabis use disorders subtype (EAPC = -0.07, 95%CI: -0.10, -0.04), upward trends of ASDR were observed in the other subtypes (Additional File 1: Table S4; Additional File 2: Figure S1). Similar trends could be found at the global level in some subtypes, and the downward trends could be seen more widely (Additional File 1: Tables S1-S8).

## Factors influencing the EAPC in DUDs in the US

**Fig. 4:**
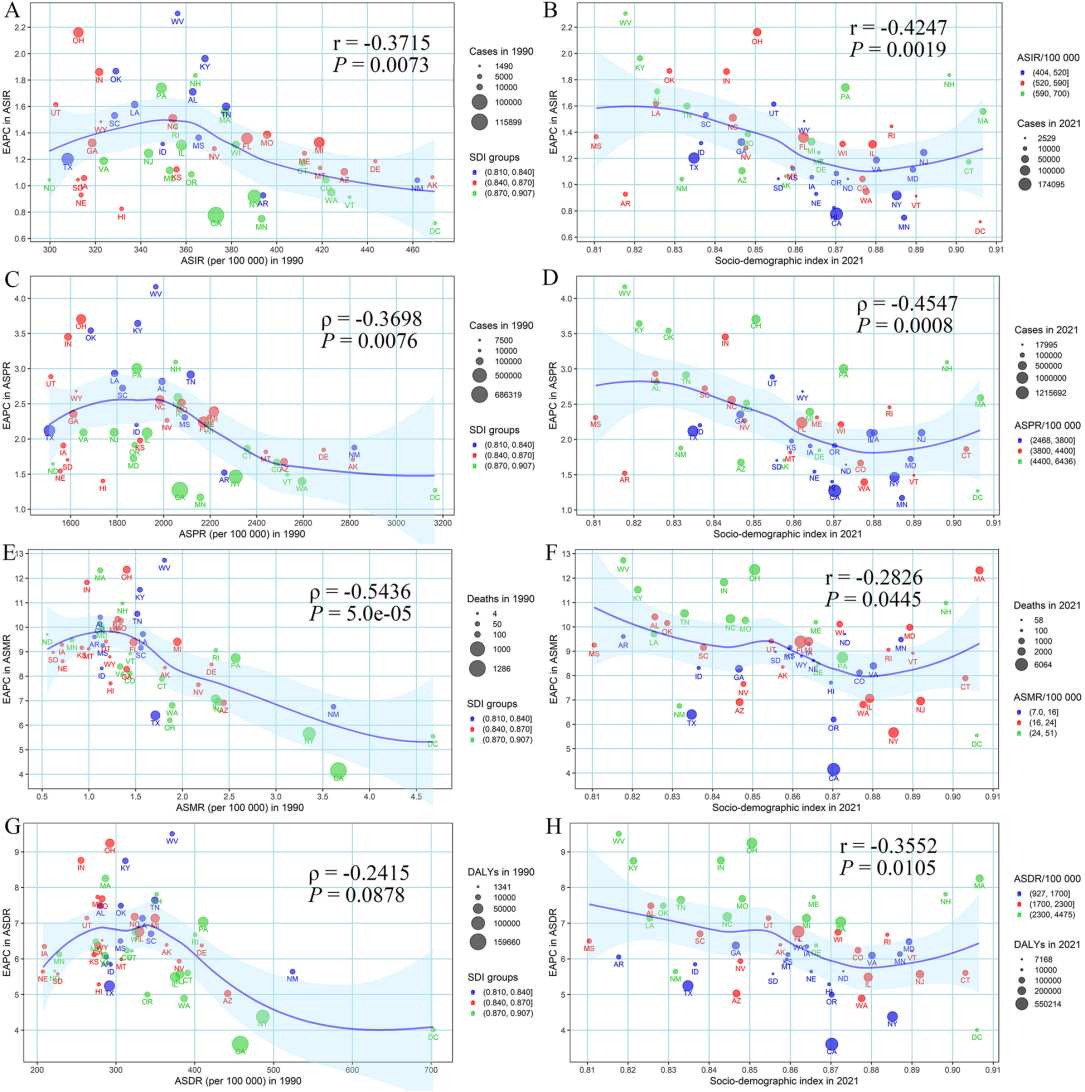
The influence factors of the EAPCs of drug use disorders-related burden in the US. **A** ASIR in 1990 and EAPC in ASIR; **B** SDI in 2021 and EAPC in ASIR; **C** ASPR in 1990 and EAPC in ASPR; **D** SDI in 2021 and EAPC in ASPR; **E** ASMR in 1990 and EAPC in ASMR; **F** SDI in 2021 and EAPC in ASMR; **G** ASDR in 1990 and EAPC in ASDR; **H** SDI in 2021 and EAPC in ASDR. The circle represented the state, and the size of the circle represented the number. The r or ρ indices and *P* values were evaluated by Pearson or Spearman rank analysis. The blue line and its shade were fitted by LOESS. ASIR: age-standardized incidence rate; ASPR: age-standardized prevalence rate; ASMR: age-standardized mortality rate; ASDR: age-standardized DALYs rate; EAPC: estimated annual percentage change; SDI: socio-demographic index

At the state level, a significant inverse association was identified between EAPC and DUDs burden in 1990 (*r* = -0.3715, *P* = 0.0073 for ASIR; *ρ* = -0.3698, *P* = 0.0076 for ASPR; *ρ* = -0.5436, *P* = 5.0e-5 for ASMR; and *ρ* = -0.2415, *P* = 0.0878 for ASDR, respectively) (Fig. 4A, 4C, 4E, and 4G). Meanwhile, inverse associations between age-standardized rates of EAPCs and SDI were seen in 2021 (*r* = -0.4247, *P* = 0.0019 for ASIR; *ρ* = -0.4547, *P* = 0.0008 for ASPR; *r* = -0.2826, *P* = 0.0445 for ASMR; and *r* = -0.3552, *P* = 0.0105 for ASDR, respectively), with an approximate “U” shape (turning point around 0.88) (Fig. 4B, 4D, 4F, and 4H).

### Prediction of DUDs-related burden in the US in the next 14 years

**Fig. 5:**
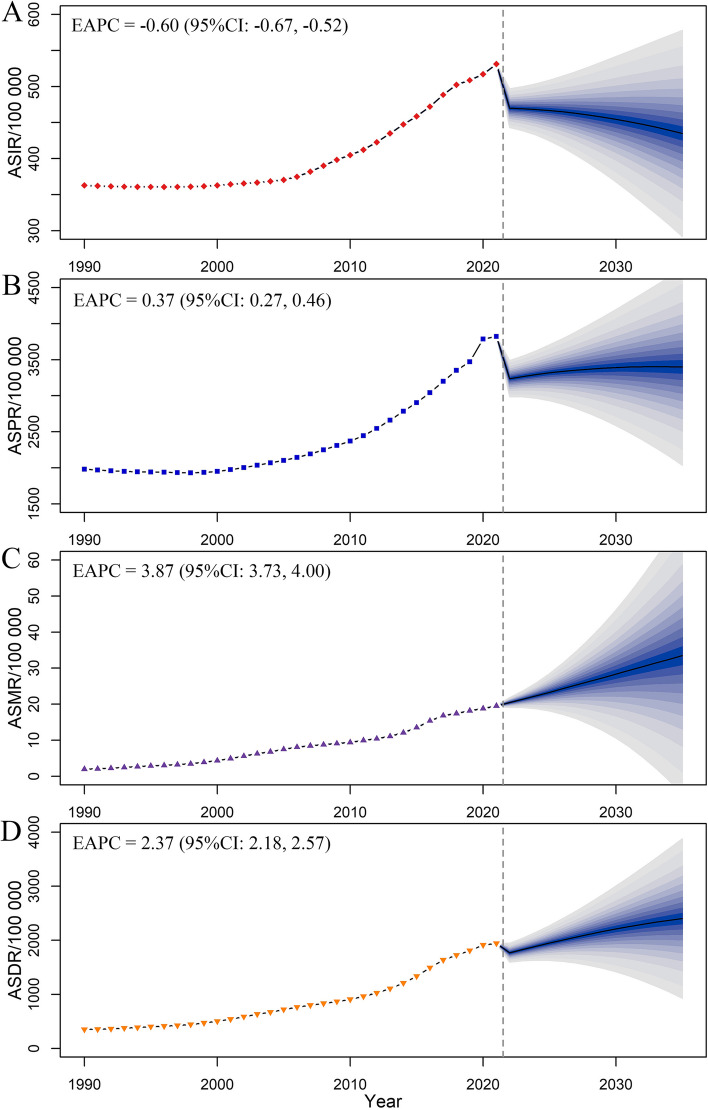
Changing trend and prediction rate of drug use disorders-related burden from 2022 to 2035 in the US. **A** ASIR from 1990 to 2035; **B** ASPR from 1990 to 2035; **C** ASMR from 1990 to 2035; **D** ASDR from 1990 to 2035. ASIR: age-standardized incidence rate; ASPR: age-standardized prevalence rate; ASMR: age-standardized mortality rate; ASDR: age-standardized DALYs rate. Shading represented a 1% decrease and increase interval based on the rate of 2021

Using the DUDs, we further predicted the burden in the next 14 years. We found that by 2035, the numbers of DUDs-related prevalent cases, deaths, and DALYs would increase to 12,241.20 thousand, 120.56 thousand, and 8653.48 thousand, representing increases of 0.78%, 70.07%, and 33.44% compared to 2021, respectively; while the number of incident cases would slightly decrease by 1.09% (with the number of 1566.16 in 2035) (Additional File 1: Table S9). Meanwhile, from 2022 to 2035, the ASIR (EAPC = -0.60, 95%CI: -0.67, -0.52) would decrease; however, the ASPR (EAPC = 0.37, 95%CI: 0.27, 0.46), ASMR (EAPC = 3.87, 95%CI: 3.73, 4.00), and ASDR (EAPC = 2.37, 95%CI: 2.18, 2.57) would increase (Fig. 5).

## Discussion

This study disclosed the magnitude and temporal trends of DUDs-related burden during the past three decades in the US based on the lasted GBD 2021 and also conducted the projection until 2035. It revealed that the absolute number of DUDs in the US accounted for a relatively high proportion of the global numbers in 2021, especially the number of deaths (accounting for 51.6%). As measured by trends, the burden of DUDs in the US increased significantly from 1990 to 2021. However, during the same period, a slight increase was only observed in ASMR worldwide. DUDs have become a serious disease burden in the US, and more targeted health policies and projects should be timely taken to alleviate this burden.

From the perspective of drug categories, opioid use disorders had the heaviest burden in the US in 2021. Especially, ASPR, ASMR, and ASDR of opioid use disorders accounted for over 50% of all types of DUDs. Given that the number of deaths from opioid overdoses reached a historic high in 2016, the US Department of Health and Human Services (HHS) declared the opioid crisis a public health emergency in 2017 [22, 23]. As of 2020, 37.309 million Americans aged 12 and above had used illegal drugs in the past 30 days, of which 24.7% had opioid use disorders [9]. Our study showed that in 2021, opioids accounted for more than 78% of the deaths attributed to DUDs in the US, exceeding half of the global death toll. In the same year, the ASMR of opioid use disorders in the US was 12.92 times higher than in the world. Opioids remain the most lethal drug, notably, strategies have been implemented to control the burden caused by opioid use disorders. Buprenorphine, methadone, and naltrexone have been approved by the FDA respectively for the treatment of opioid dependence, they are proven to be safe and effective when combined with counseling and behavioral therapies, known as medication-assisted treatment (MAT) [24]. The FDA subsequently approved the first over-the-counter (OTC) naloxone nasal spray and the first namefene hydrochloride nasal spray in March and May 2023, respectively [25]. These drugs belong to opioid receptor antagonists and can be used to treat acute opioid overdose, thereby reducing opioid overdose mortality. Our research findings also suggest that the implementation of these strategies has achieved certain effects, the overall ASIR will decrease in the next 14 years. Although the ASPR, ASMR, and ASDR would increase, with the implementation of strategies, they would also decrease.

DUDs in the US were more severe among males than females in most age-specific groups in 2021, possibly due to males being more likely to receive higher doses of psychotropic drugs [26]. There are also reports showing that during the previous year, males in the US were 2.33 and 2.25 times more likely than females to suffer from DUDs and drug dependence, respectively [27, 28]. In addition, the number of death cases caused by DUDs among females has been consistently high from age groups of 25 to 59, and the peak age group appears later than males. This may be related to females who use psychotropic drugs later and are more prone to mental disorders compared to males, or there are more obstacles for females in accessing medication, leading to insufficient medication treatment [9, 29, 30]. The comprehensive women-centered treatment method includes treating the whole person and the mother–child dyad [31]. This may consist of a variety of intervention measures and services, such as childcare and parenting education; obstetric and gynecological care; general medical care; interventions and services for comorbid mental disorders; social support, including transportation, housing, and occupational rehabilitation; and legal aid [31]. However, females with DUDs have to face more pressure and barriers when receiving the treatment, they may endure more social stigmatization, fear legal sanctions, and possibly even lose custody of their children [9, 32]. More practical and effective strategies for women should also be developed and implemented to alleviate or even relieve burdens.

Regardless of gender, the burden of DUDs is highest in the age-specific groups of 15–45 years and showed a decreasing trend with age, which was consistent with the world pattern. This indicated that in young adults DUDs were still very serious, especially in youthfulness. Studies have estimated that the past year and lifetime prevalence of DUDs among 18–29-year-olds in the US is 8.3% and 14.2%, respectively [33]. The earlier the use of psychoactive drugs, the greater the lifelong risk of DUDs [34, 35]. Among users who try psychotropic drugs before the age of 13, 70% develop DUDs within the next 7 years, while for those after the age of 17, the proportion drops to 27% [36]. These adolescents and children can suffer physical, sexual, and emotional abuse, and be in a state of poverty, homelessness, famine, and gender discrimination [36, 37]. Due to the neurotoxicity of psychoactive drugs on the developing brain, contact with the drugs should be immediately interrupted to reduce the damage [3]. Meanwhile, treatment services for DUDs should be provided promptly to accurately identify and meet the needs of adolescents and children [38]. In recent years, treatments including but not limited to cognitive behavioral therapies, vocational training, psychoeducation, and motivational enhancement therapies, have been applied across service settings in the US [3, 39–41]. These approaches have achieved certain benefits in treating adolescent DUDs, as shown in this study, age-specific rates of incidence, prevalence, and DALYs have all decreased in the 10–14 age group.

Our results suggested that in 2021, the EAPCs for DUDs in various states in the US were negatively correlated with the overall SDI. Especially in West Virginia, it has lower SDI but in most cases higher EAPC, indicating a heavy drug abuse burden. According to reports, West Virginia has the highest per capita opioid overdose mortality rate and the highest case rate of neonatal opioid withdrawal (NOW) syndrome in the US [36]. Since the early 2010s, several states in the US have begun strengthening prescription drug monitoring programs (PDMP), with dramatic results, for example, Kentucky reduced its number of opioid prescriptions per capita by 85% in 2015 compared with 2010, Florida saw a 50% drop in oxycodone overdose deaths in 2012 compared with 2010, national opioid prescriptions fell 16% annually, and the rate of Americans receiving MAT increased 13% annually [36]. It should also be noted that the burden of DUDs usually increases with increasing SDI values [1]. People from higher SDI areas have relatively higher socio-economic status, they may have more propensity to initiate drugs [31]. However, populations in lower SDI areas may face more socio-economic disadvantages such as poverty, conflict, and lack of education and employment opportunities, making them highly susceptible to mental health issues and DUDs, as well as limited access to health promotion, prevention, and drug treatment services. Therefore, the latter pays a higher price and is highly likely to suffer a heavy burden [31].

Despite previous reports on the limitations of GBD [10–12], it is still necessary to clarify the limitations of this work. Firstly, in the GBD study (2021), the DUDs were defined based on the DSM-IV-TR or the ICD-10 diagnostic criteria, if the Diagnostic and Statistical Manual of Mental Disorders (DSM-5) was used, the estimation of DUDs may be changed. Secondly, the limited detailed information obtained from the GBD database regarding the US and its states in this study may limit the analysis of the burden of DUDs, although GBD study (2021) made improvements to the model to improve estimation accuracy. In addition, due to the lack of estimated deaths caused by cannabis use disorders in the GBD 2021 data, the total number of deaths from DUDs may be underestimated. Last but not least, this study evaluated the changing trends and predicted the burden of DUDs based on the GBD study 2021, but due to the information lag in this database (only data information from 1990 to 2021 could be obtained currently), the prediction results may not be accurate enough. However, our results still have significant public health implications for controlling the DUDs-related burden in the US.

## Conclusions

In conclusion, in the past three decades, the burden of DUDs in the US was heavy, especially in males, 15–45 years old populations, and opioid use disorders subtype. From 1990 to 2021, the DUDs-related burden increased in the US and all states, especially in West Virginia. In the next 14 years, the projected DUDs burden remains exigent. Therefore, our study proposes that greater attention should be paid to the younger population, males and older females, opioid use disorders, and low-SDI states implemented by health policymakers to achieve goals such as reducing burden.

### Supplementary Information

Additional File 1: Supplementary tables S1-S10. Table S1. Number of incident cases and incidence rate of drug use disorders in the US in 1990 and 2021, and the temporal trends from 1990 to 2021. Table S2. Number of prevalent cases and prevalence rate of drug use disorders in the US in 1990 and 2021, and the temporal trends from 1990 to 2021. Table S3. Number of death cases and mortality rate of drug use disorders in the US in 1990 and 2021, and the temporal trends from 1990 to 2021. Table S4. Number of DALYs cases and DALYs rate of drug use disorders in the US in 1990 and 2021, and the temporal trends from 1990 to 2021. Table S5. Number of incident cases and incidence rate of global drug use disorders in 1990 and 2021, and the temporal trends from 1990 to 2021. Table S6. Number of prevalent cases and prevalence rate of global drug use disorders in 1990 and 2021, and the temporal trends from 1990 to 2021. Table S7. Number of death cases and mortality rate of global drug use disorders in 1990 and 2021, and the temporal trends from 1990 to 2021. Table S8. Number of DALYs cases and DALYs rate of global drug use disorders in 1990 and 2021, and the temporal trends from 1990 to 2021. Table S9. Prediction number of drug use disorders-related burden from 2022 to 2035 in the US. Table S10. Number and rate of drug use disorders burden by state, sex, and type in the US in 2021, and the temporal trends from 1990 to 2021.

Additional File 2: Supplementary figures S1-S3. Figure S1. Numbers and age-standardized rates of drug use disorders-related burden by sex and subtype from 1990 to 2021 in the US. (A) numbers and age-standardized rates of incidence; (B) numbers and age-standardized rates of prevalence; (C) numbers and age-standardized rates of deaths; and (D) numbers and age-standardized rates of DALYs. The bar plots represented the numbers; the line plots and their shade represented the age-standardized rates and their 95%UIs. DALYs: disability-adjusted life years; UI: uncertainty interval. Figure S2. Age-specific numbers of drug use disorders-related burden by sex from 1990 to 2021 in the US and the world. (A) age-specific numbers of incident cases; (B) age-specific numbers of prevalent cases; (C) age-specific numbers of deaths; and (D) age-specific numbers of DALYs. DALYs: disability-adjusted life years. Figure S3. Age-specific rates of drug use disorders-related burden by sex from 1990 to 2021 in the US and the world. (A) age-specific rates of incidence; (B) age-specific rates of prevalence; (C) age-specific rates of deaths; and (D) age-specific rates of DALYs. DALYs: disability-adjusted life years.

## Data Availability

Data can be obtained from the following website: http://ghdx.healthdata.org/gbd-results-tool.

## Abbreviations

ASDR: Age-standardized DALYs rate
ASIR: Age-standardized incidence rate
ASMR: Age-standardized mortality rate
ASPR: Age-standardized prevalence rate
CI: Confidence interval
CODEm: Cause of Death Ensemble model
DALYs: Disability-adjusted life years
EAPC: Estimated annual percentage change
GBD: The Global Burden of Diseases, Injuries, and Risk Factors Study
LMER: Linear mixed effects regression
ST-GPR: Spatiotemporal Gaussian process regression
UI: Uncertainty interval
WHO: World Health Organization
YLDs: Years lived with disability
YLLs: Years of life lost
