# Supplementary material for: Burden of drug use disorders in the United States from 1990 to 2021 and its projection until 2035: results from the GBD study
Source: BMC Public Health. 2024 Jun 19;24:1639. doi: 10.1186/s12889-024-19142-0 (PMC11188227; doi:10.1186/s12889-024-19142-0)
Supplement: Supplementary file 2 — Additional File 2: Supplementary figures S1-S3. Figure S1. Numbers and age-standardized rates of drug use disorders-related burden by sex and subtype from 1990 to 2021 in the US. (A) numbers and age-standardized rates of incidence; (B) numbers and age-standardized rates of prevalence; (C) numbers and age-standardized rates of deaths; and (D) numbers and age-standardized rates of DALYs. The bar plots represented the numbers; the line plots and their shade represented the age-standardized rates and their 95%UIs. DALYs: disability-adjusted life years; UI: uncertainty interval. Figure S2. Age-specific numbers of drug use disorders-related burden by sex from 1990 to 2021 in the US and the world. (A) age-specific numbers of incident cases; (B) age-specific numbers of prevalent cases; (C) age-specific numbers of deaths; and (D) age-specific numbers of DALYs. DALYs: disability-adjusted life years. Figure S3. Age-specific rates of drug use disorders-related burden by sex from 1990 to 2021 in the US and the world. (A) age-specific rates of incidence; (B) age-specific rates of prevalence; (C) age-specific rates of deaths; and (D) age-specific rates of DALYs. DALYs: disability-adjusted life years. [file 12889_2024_19142_MOESM2_ESM.docx]

Burden of drug use disorders in the United States from 1990 to 2021 and its projection until 2035: results from the GBD study

Running title: Drug use burden in the US, 1990-2035.

Tongchao Zhang ^1,2,#^; Lin Sun ^3,#^; Xiaolin Yin ^1,2^; Hui Chen ^1,2^; Lejin Yang ^4^; Xiaorong Yang ^1,2,*^

1 Clinical Epidemiology Unit, Qilu Hospital of Shandong University, Jinan, China.

2 Clinical Research Center of Shandong University, Jinan, China.

3 Department of Pharmacy, Qilu Hospital of Shandong University, Jinan, China.

4 Department of Psychology, Qilu Hospital of Shandong University, Jinan, China.

^#^ Tongchao Zhang and Lin Sun contributed equally to this work.

* Corresponding Author: Xiaorong Yang, Clinical Epidemiology Unit, Qilu Hospital of Shandong University, 107 Wenhuaxi Road, Jinan, Shandong 250012, China. Email: yangxiaorong@sdu.edu.cn

Additional File 2: Supplementary figures S1-S3

Figure S1. Numbers and age-standardized rates of drug use disorders-related burden by sex and subtype from 1990 to 2021 in the US. (A) numbers and age-standardized rates of incidence; (B) numbers and age-standardized rates of prevalence; (C) numbers and age-standardized rates of deaths; and (D) numbers and age-standardized rates of DALYs. The bar plots represented the numbers; the line plots and their shade represented the age-standardized rates and their 95%UIs. DALYs: disability-adjusted life years; UI: uncertainty interval.

Figure S2. Age-specific numbers of drug use disorders-related burden by sex from 1990 to 2021 in the US and the world. (A) age-specific numbers of incident cases; (B) age-specific numbers of prevalent cases; (C) age-specific numbers of deaths; and (D) age-specific numbers of DALYs. DALYs: disability-adjusted life years.

Figure S3. Age-specific rates of drug use disorders-related burden by sex from 1990 to 2021 in the US and the world. (A) age-specific rates of incidence; (B) age-specific rates of prevalence; (C) age-specific rates of deaths; and (D) age-specific rates of DALYs. DALYs: disability-adjusted life years.


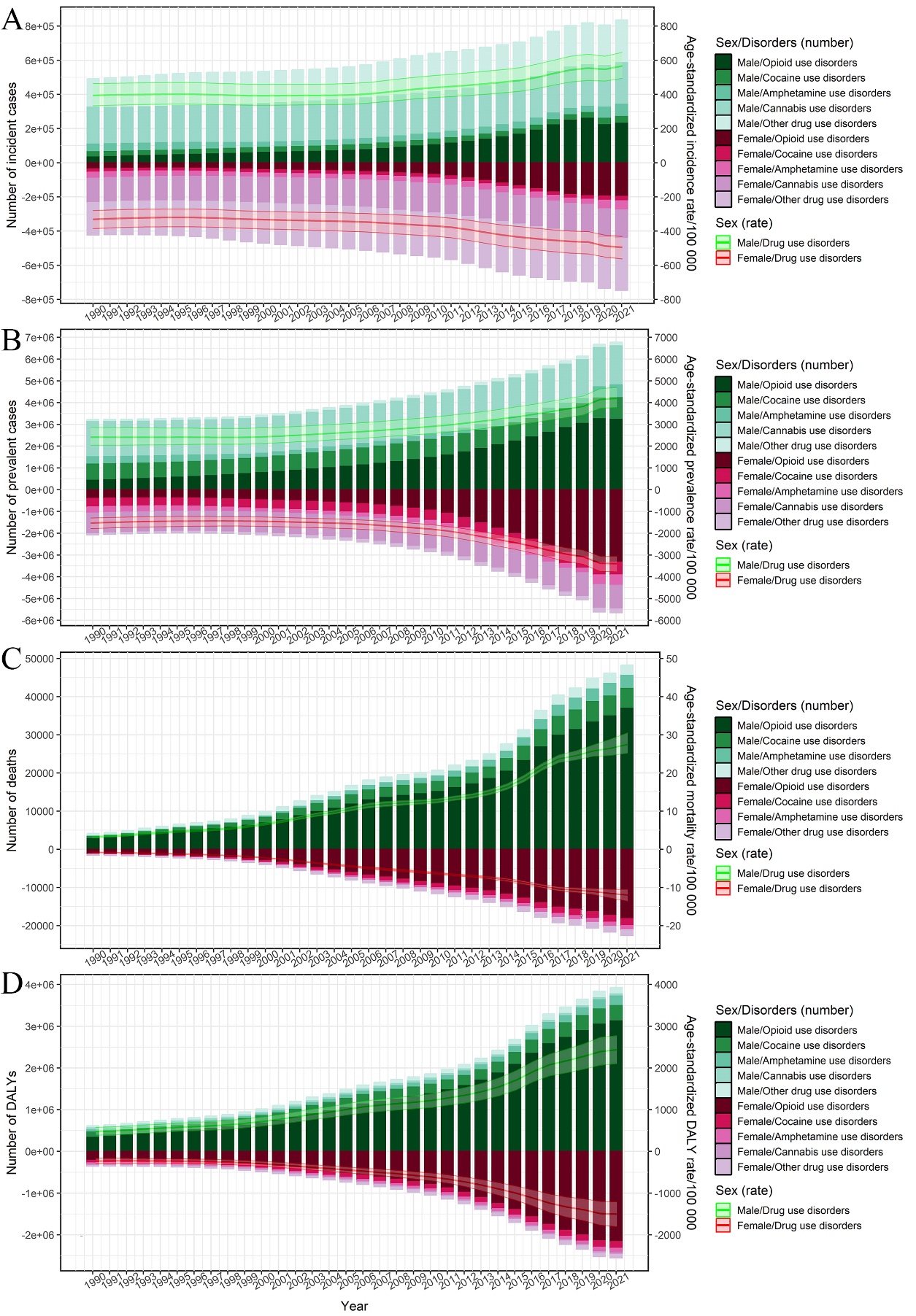


Figure S1. Numbers and age-standardized rates of drug use disorders-related burden by sex and subtype from 1990 to 2021 in the US. (A) numbers and age-standardized rates of incidence; (B) numbers and age-standardized rates of prevalence; (C) numbers and age-standardized rates of deaths; and (D) numbers and age-standardized rates of DALYs. The bar plots represented the numbers; the line plots and their shade represented the age-standardized rates and their 95%UIs. DALYs: disability-adjusted life years; UI: uncertainty interval.


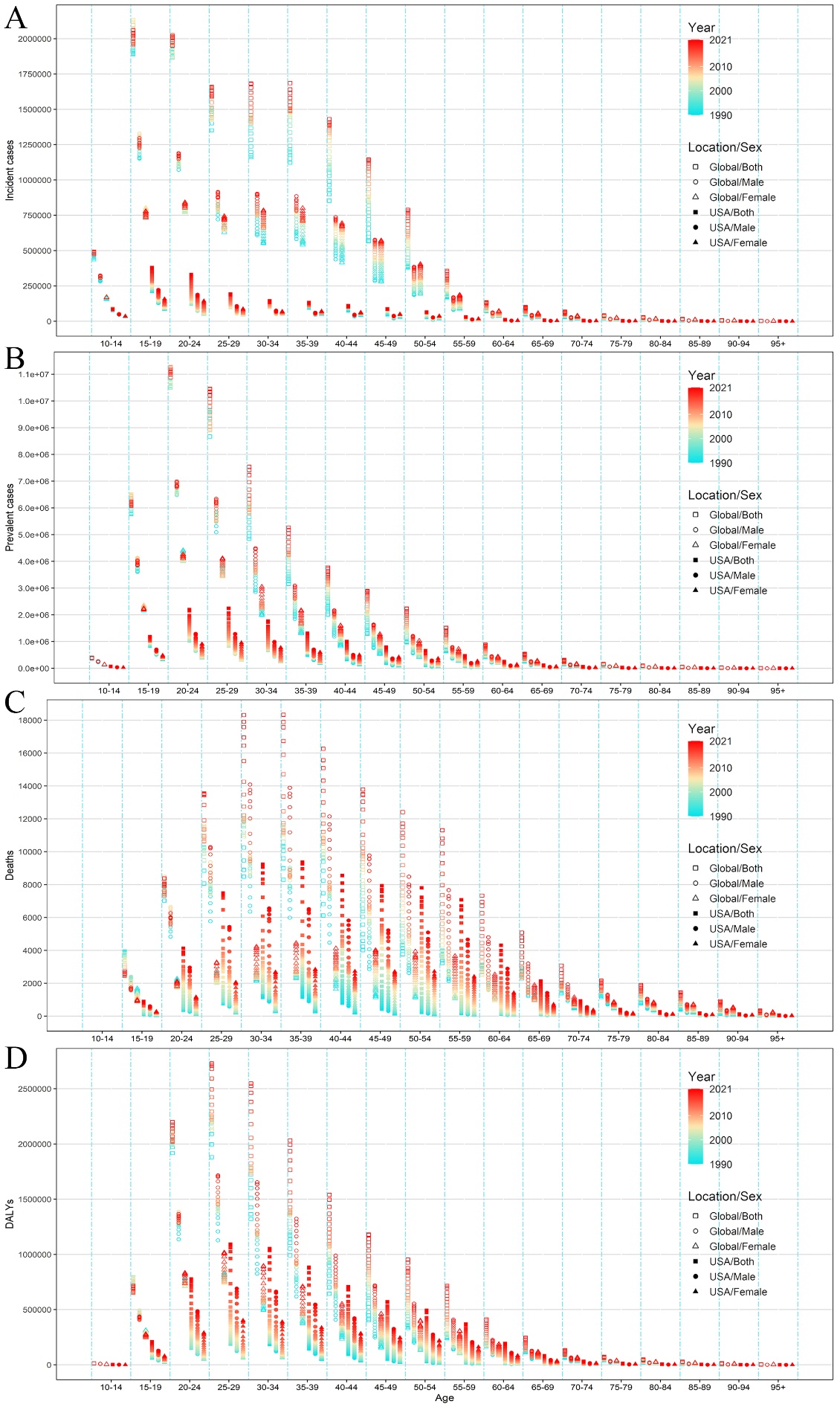


Figure S2. Age-specific numbers of drug use disorders-related burden by sex from 1990 to 2021 in the US and the world. (A) age-specific numbers of incident cases; (B) age-specific numbers of prevalent cases; (C) age-specific numbers of deaths; and (D) age-specific numbers of DALYs. DALYs: disability-adjusted life years.


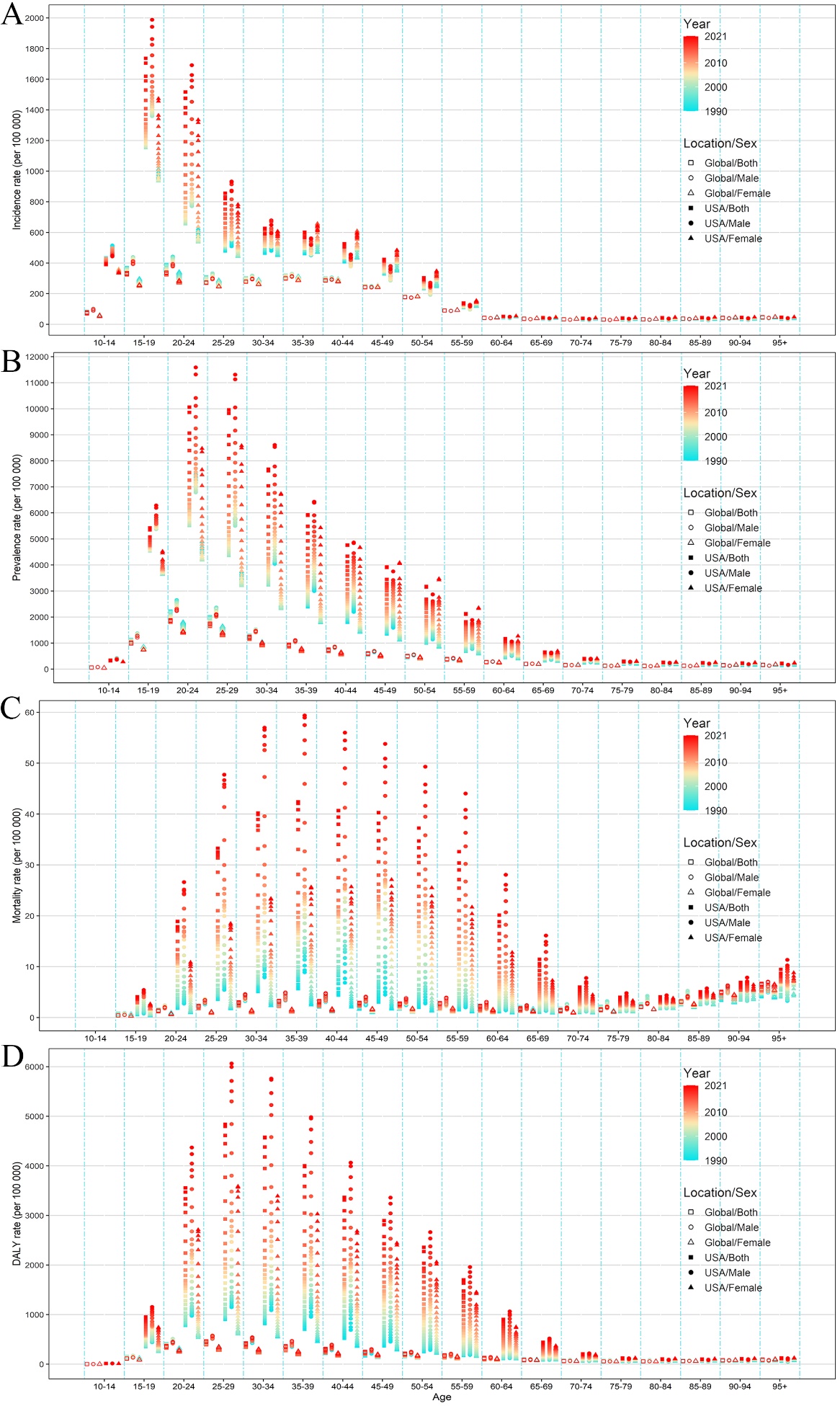


Figure S3. Age-specific rates of drug use disorders-related burden by sex from 1990 to 2021 in the US and the world. (A) age-specific rates of incidence; (B) age-specific rates of prevalence; (C) age-specific rates of deaths; and (D) age-specific rates of DALYs. DALYs: disability-adjusted life years.
